# Mucinous Cystadenoma of Ovary with Vague Symptoms: A Case Report

**DOI:** 10.31729/jnma.7513

**Published:** 2022-08-31

**Authors:** Paras Khadayat, Bhagirathi Kayastha, Prashant Koirala

**Affiliations:** 1 Kathmandu University School of Medical Sciences, Dhulikhel, Kavre, Nepal; 2 Department of Gynecology and Obstetrics, Kathmandu University School of Medical Sciences, Dhulikhel, Kavre, Nepal

**Keywords:** *case report*, *ovarian cysts*, *symptoms*

## Abstract

Ovarian cyst is a fluid-filled sac in the ovary, common among reproductive women. Mucinous cystadenoma is a common variety of non-functional benign lesions that presents vague symptoms and can mislead the diagnosis. A 26-year-old female presented to the clinic with weakness and lethargy, which lasted for 3 months, along with other symptoms like headache, abdominal pain, bloating, nausea, and constipation. She was managed with iron deficiency anaemia, however, her symptoms did not improve. Later, an ultrasound revealed an ovarian cyst. Laparoscopic left-sided ovarian cystectomy was performed and a biopsy was sent for histopathological examination. The case highlights the various nonspecific symptoms in a case of an ovarian cyst. Gynaecological causes for systemic symptoms should always be considered, along with proper gynaecological history and examination. This helps with the accuracy of diagnosis and treatment options, with minimal costs.

## INTRODUCTION

An ovarian cyst is a fluid-filled sac in the ovary, common in reproductive age. Functional cysts are common.^1^ These are usually asymptomatic and self-limiting, requiring no or fewer interventions.^2^ But neoplastic lesions, benign or malignant, produce vague symptoms due to mass and pressure effects in surrounding organs.^3^ This case report describes a patient with an ovarian cyst presented with complaints of various nonspecific and vague symptoms such as weakness, lethargy, headache, constipation, and vomiting. She was treated with iron deficiency anaemia. Along with comprehensive gynaecological history and examination, gynaecological causes for any systemic symptoms should always be taken into consideration.

## CASE REPORT

A 26-year-old unmarried female presented to the outpatient department (OPD) with complaints of tiredness, lethargy, and weakness for the past 3 months. She also complained about abdominal pain, bloating, constipation, nausea, and headache on and off during this period. There was no similar illness in the past or any family members. She did not smoke, consume alcohol or took any medication.

On the first day of the presentation, a general examination was performed and vitals were measured, which were found to be within physiological limits. A thorough gynaecological history was overlooked and was not addressed on time. Several exams were performed: hemogram, thyroid function test, serum iron level, C-reactive protein, liver function test, lipid profile, folic acid, active vitamin B12, and Vitamin D levels. On the second day, the result showed haemoglobin was marginally decreased to 11.7 mg/dl with decreased serum iron (4.2 μmol/l) and active B12 which was 24 pmol/l while all other parameters were in a normal range. She was diagnosed as iron deficiency anaemia and vitamin B12 deficiency due to inadequate dietary intake. She has been prescribed 325 mg iron supplement tablets thrice daily, and 100 mcg vitamin B12 tablets twice daily and she was scheduled for a follow-up in 2 months.

**Figure 1:**
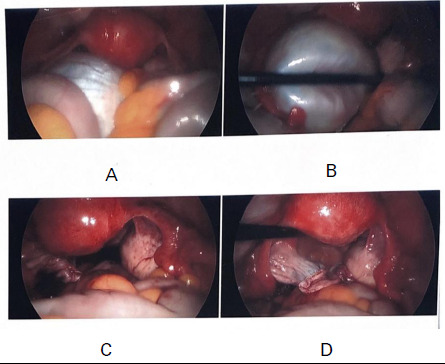
Laparoscopic removal of left ovarian cyst A, B) Before removal, C, D) After removal.

After 2 months, there was no improvement in symptoms, and haemoglobin increased by 0.1 mg/dl despite iron and vitamin supplementation. Later, the physician ordered tests to find the cause of decreased synthesis of blood. Tests such as parietal cell antibodies and intrinsic factor antibodies also provided negative results. The blood smear shows microcytic anaemia with increased reticulocyte counts indicating chronic blood loss. Suspicion for increased blood loss causing iron deficiency anaemia rather than decreased synthesis was proposed. On taking menstruation history, she gave the history of heavy bleeding occurring for 6-7 days with the passage of clots during menstruation for 8 months without realising it was a symptom of pathology, and not having a gynaecological consultation for it. Her last menstruation period was 7 days ago with a regular cycle of 30 days. Ultrasound of the pelvis and X-ray of the abdomen was ordered to find gynaecological cause for heavy bleeding during menstruation. Ultrasound of the pelvis revealed a cyst on the left ovary measuring 10 × 6.1 × 7.4 cm with no internal vascularity or any solid component. There was no adnexal mass or fluid in Cul-de-sac. Tumour biomarkers CA-125 and CA-19.9 were sent to estimate malignancy potential and were 36 U/ml and 45 U/ml respectively. She was referred to a gynaecologist for further management and counselled about treatment options, outcomes and prognosis. As the size of the cyst was large, it could undergo torsion or rupture. Also when considering the patient's young age and potential future fertility minimal access to laparoscopic surgery was advised and performed after 1 week. Laparoscopic left-sided ovarian cystectomy was performed and a biopsy was sent for histopathological examination (Figure 1).

Operative findings included a large left-sided ovarian cyst and a smooth surface without breach. Enucleation of the cyst was done and later the cyst was punctured and thick viscous material was suctioned out. The contralateral ovary and bilateral tubes were normal. The histopathological report showed left ovarian benign mucinous cystadenoma.

The postoperative period was uneventful and the patient was discharged after 48 hours. She came to a follow-up after 1 week and reported no complaints.

## DISCUSSION

Mucinous cystadenoma is one of the common tumours that account for about 15-20% of all ovarian neoplasms. About 80% of mucinous tumours are benign and occur mostly in women of reproductive age.^4^ Risk factors for ovarian cysts include; pregnancy, hypothyroidism, smoking, infertility treatment, etc.^1^ In many cases, they remain undiagnosed as they do not produce symptoms at all. Whereas, in others, clinical signs and symptoms of the cyst are non-specific and vague making diagnosis difficult.^5^ According to the study, the most common presenting symptom in ovarian neoplasm is abdominal pain followed by abdominal distension, urinary complaints, vaginal discharge, and bleeding.^6^ Rare complications of ovarian cyst are torsion, rupture and haemorrhage. But, in our case, the patient presented with menorrhagia which results in iron deficiency anaemia. Heavy bleeding during the menstrual cycle is one of the commonest causes of iron deficiency anaemia in reproductive women.^7^

Ultrasonography plays an important role in the diagnosis of benign or malignant lesions of pelvis and evaluation of menorrhagia.^8^ Oral contraceptive pills are given for the treatment of ovarian cysts but they are not found to be effective in various trials.^9^ However, Oral contraceptive pills are effective in the prevention of the development of cysts. Most ovarian cysts do not require any treatment and undergo spontaneous regression after a few cycles. Surgical removal is indicated only if it persists for a longer time, is larger in size, symptomatic. Treatment of mucinous cystadenoma is laparotomy or laparoscopic surgery depending on various factors like size of the cyst, age of the patient, parity, clinical presentation, etc.^10^ In our case, the patient underwent laparoscopic surgery as it has diagnostic and therapeutic value. Moreover, the laparoscopic technique is the better option over conventional laparotomy as it reduces blood loss, pain, morbidity/mortality, and hospital stay and has a cosmetically better scar.^11^ The prognosis of benign mucinous cystadenoma is usually good with a high survival rate when treated timely.^12^

This case explains the vague symptoms of the ovarian cyst. It also reflects the importance of detailed gynaecological history in the diagnosis of pathology and treatment plan options. The gynaecological problems can be missed. The gynaecological cause for the systemic illness must be ruled out when the patient presents with non-gynaecological symptoms by the treating physician. Proper history taking and physical examination help to limit our differential diagnosis and reduce the need for unnecessary testing and delay in treatment.
